# Pre-to-post diagnosis weight trajectories in colorectal cancer patients with non-metastatic disease

**DOI:** 10.1007/s00520-018-4560-z

**Published:** 2018-11-27

**Authors:** Moniek van Zutphen, Anouk Geelen, Hendriek C. Boshuizen, Renate M. Winkels, Anne J.M.R. Geijsen, Evertine Wesselink, Merel Snellen, Dieuwertje E. Kok, Johannes H.W. de Wilt, Paul C. van de Meeberg, Ewout A. Kouwenhoven, Henk K. van Halteren, Ernst J. Spillenaar Bilgen, Ellen Kampman, Fränzel J.B. van Duijnhoven

**Affiliations:** 1 Division of Human Nutrition and Health, Wageningen University and Research, Stippeneng 4, 6708 WE Wageningen, The Netherlands; 2 Department Public Health Sciences, College of Medicine, Penn State University, Hershey, PA USA; 3 Department of Surgery, Radboud University Medical Centre, Geert Grooteplein-Zuid 22, 6525 GA Nijmegen, The Netherlands; 4 Department of Gastroenterology, Slingeland Hospital, P.O. Box 169, 7000 AD Doetinchem, The Netherlands; 5 Department of Surgery, Hospital Group Twente ZGT, Zilvermeeuw 1, 7609 PP Almelo, The Netherlands; 6 Department of Internal Medicine, Admiraal de Ruyter Ziekenhuis, ‘s-Gravenpolderseweg 114, 4462 RA Goes, The Netherlands; 7 Department of Surgery, Rijnstate Hospital, Wagnerlaan 55, 6815 AD Arnhem, The Netherlands

**Keywords:** Colorectal cancer, Weight change, Weight gain, Chemotherapy

## Abstract

**Purpose:**

Previous studies have shown that > 50% of colorectal cancer (CRC) patients treated with adjuvant chemotherapy gain weight after diagnosis. This may affect long-term health. Therefore, prevention of weight gain has been incorporated in oncological guidelines for CRC with a focus on patients that undergo adjuvant chemotherapy treatment. It is, however, unknown how changes in weight after diagnosis relate to weight before diagnosis and whether weight changes from pre-to-post diagnosis are restricted to chemotherapy treatment. We therefore examined pre-to-post diagnosis weight trajectories and compared them between those treated with and without adjuvant chemotherapy.

**Methods:**

We included 1184 patients diagnosed with stages I–III CRC between 2010 and 2015 from an ongoing observational prospective study. At diagnosis, patients reported current weight and usual weight 2 years before diagnosis. In the 2 years following diagnosis, weight was self-reported repeatedly. We used linear mixed models to analyse weight trajectories.

**Results:**

Mean pre-to-post diagnosis weight change was −0.8 (95% CI −1.1, −0.4) kg. Post-diagnosis weight gain was + 3.5 (95% CI 2.7, 4.3) kg in patients who had lost ≥ 5% weight before diagnosis, while on average clinically relevant weight gain after diagnosis was absent in the groups without pre-diagnosis weight loss. Pre-to-post diagnosis weight change was similar in patients treated with (−0.1 kg (95%CI −0.8, 0.6)) and without adjuvant chemotherapy (−0.9 kg (95%CI −1.4, −0.5)).

**Conclusions:**

Overall, hardly any pre-to-post diagnosis weight change was observed among CRC patients, because post-diagnosis weight gain was mainly observed in patients who lost weight before diagnosis. This was observed independent of treatment with adjuvant chemotherapy.

## Introduction

Survival of colorectal cancer (CRC) has markedly improved over recent decades, which underlines the importance to study factors that can affect long-term health and quality of life of CRC survivors. One of the factors that may affect health and quality of life is body weight. Weight loss, either before diagnosis or during cancer treatment, is an important negative prognostic marker [1–4]. Therefore, in the hospital nutritional advice to cancer patients is mainly focused on prevention and/or treatment of unintentional weight loss. However, overweight and obesity are also affecting long-term health and quality of life among patients with non-metastatic disease. Therefore, prevention of weight gain after CRC diagnosis has recently been incorporated in the Dutch oncological nutritional therapy guidelines [5].

Many CRC patients are overweight or obese at diagnosis, as excess body weight is a risk factor for CRC [6]. Overweight/obese CRC survivors have an elevated risk of co-morbid disease, such as cardiovascular disease and diabetes, both at diagnosis and in the years following a diagnosis [7–9]. Weight gain after diagnosis might exacerbate existing co-morbid disease progression and further increase the risk of developing such diseases. Several studies reported that weight gain after diagnosis is common among CRC patients [1–3, 10, 11]. All these studies showed that weight gain after diagnosis was more common than weight loss after diagnosis [1–3, 10, 11]. The proportion of weight gain after diagnosis typically ranged from 25% to over 50% of patients [1–3, 10, 11]. In these studies, weight gain was defined as either a weight gain of ≥ 5 kg [1, 10] or ≥ 5% [2, 3, 11].

Although body weight may increase after CRC diagnosis, studies so far did not assess how body weight changed relative to usual body weight before diagnosis. Weight loss before CRC diagnosis is common [4, 12] as unintended weight loss could be one of the reasons for patients to see a physician, leading to the diagnosis of CRC. Thus, it is possible that patients catch up for this pre-diagnostic weight loss in the period during and after treatment. It is currently unknown if post-diagnosis weight change is different for patients with pre-diagnosis weight change compared to patients who were weight stable before diagnosis. Post-diagnosis weight gain might be more problematic in terms of long-term health if it results in overall weight gain compared to usual weight than when it reflects catching up for pre-diagnostic weight loss.

Weight gain is a common side-effect of chemotherapy in breast cancer patients [13], but weight gain is also common among non-metastatic CRC patients during and after chemotherapy. Two studies that both included > 500 colon cancer patients with stage III disease treated with adjuvant chemotherapy reported that the majority (51–65%) of patients experienced weight gain [3, 10]. Weight gain is observed both during and after adjuvant chemotherapy [11]. Therefore, prevention of weight gain in oncological guidelines has a focus on patients treated with adjuvant chemotherapy [5]. However, there is only indirect evidence that weight gain after diagnosis is more prevalent among patients treated with adjuvant chemotherapy than among patients treated without adjuvant chemotherapy. Studies that included non-metastatic CRC patients irrespective of chemotherapy treatment reported lower proportions (28%) of weight gain [1, 2] than studies among CRC patients treated with adjuvant chemotherapy (51–65%) [3, 10]. There are no studies that directly compared weight changes between patients treated with or without adjuvant chemotherapy.

Weight trajectories should ideally include data on weight at multiple time points, both before and after diagnosis, to fully capture weight changes among CRC patients. This information is currently lacking and therefore it remains unclear whether post-diagnosis weight eventually surpasses usual pre-diagnosis weight. Our aim was to examine pre-to-post diagnosis weight trajectories in CRC patients with non-metastatic disease and to compare these weight trajectories among patients treated with and without adjuvant chemotherapy.

## Methods

### Study population

We used data of the COLON study, an ongoing prospective multicentre cohort study among CRC patients in the Netherlands [14]. Eligible participants with newly diagnosed colon or rectal cancer were invited by hospital staff to participate in the study during a routine clinical visit before scheduled surgery. Data were collected shortly after diagnosis, before treatment started, and at two or three time points in the first 2 years after diagnosis (see “Assessment of body weight”). Follow-up data were available until January 2018. All study participants provided written informed consent and the study was approved by the local review board.

This study was performed among all participants diagnosed with stage I–III CRC between 2010 and 2015 who had a surgical resection (*n* = 1225). We excluded 70 participants who had information on weight available for < 2 time points. Thus, data of 1152 participants remained for analyses. Of these participants, 16 (1%) had missing self-reported weight before diagnosis and 217 (19%) did not complete 2 years of follow-up. We chose to exclude patients with stage IV disease a priori, because survival for these patients is generally poor and weight loss and cachexia are common at the end of life.

### Assessment of body weight

At diagnosis, participants completed a survey with questions on body weight 2 years prior to diagnosis, and current weight. Participants repetitively answered surveys about their current body weight at 6 months, 1 year (only for the subsample treated with adjuvant chemotherapy), and 2 years after diagnosis.

### Assessment of covariates

We obtained information on clinical factors, including disease stage, tumour site, receipt of neo-adjuvant treatment, type of surgery, stoma placement after surgery, complications within 30 days after surgery, receipt of adjuvant chemotherapy, type of chemotherapy, and presence of comorbidities from the Dutch ColoRectal Audit [15]. At diagnosis, all participants completed a questionnaire on demographic and lifestyle information, including education, smoking behaviour, and height. Body mass index (BMI) at diagnosis was computed in kg/m^2^.

### Statistical analyses

We calculated pre-diagnosis, post-diagnosis, and pre-to-post diagnosis weight changes as weight at the end of the period minus weight at the start of the period, so negative differences indicate weight loss and positive differences indicate weight gain. Pre-diagnostic weight changes were grouped in three pre-defined categories: weight loss ≥ 5%, weight stable −5 to +5%, and weight gain ≥ 5%. Characteristics of the study population were compared across pre-diagnosis weight change groups and across adjuvant chemotherapy treatment. Differences in categorical variables were assessed by using a chi-squared test, and differences in means of continuous variables were tested by using analysis of variance or a t-test.

We fitted linear mixed models to examine weight trajectories over 4 years (2 years pre-diagnosis to 2 years post-diagnosis). Linear mixed models take into account both the individual trajectories of change (random effects) and population averages (fixed effects) by using all available measurements and including participants with incomplete data [16]. Time was scaled in years (continuous) with the date of study enrolment (shortly after diagnosis) defined as time is zero. Time for each post-diagnosis weight was calculated as date of self-reported weight collection minus the date of study enrolment. Time for pre-diagnosis weight was set at −2 years for all subjects.

The final model included a random intercept, a random slope for time, and a random curvature for time (i.e. taking into account each participant’s weight at diagnosis and the linear and quadratic slope). Using a step-up model building strategy, the random curvature model had much better fit than a random intercept model and a random slope model.

As fixed factors, we included baseline demographic determinants (sex, age, height, education, and smoking) and clinical factors (stage, tumour site, neo-adjuvant treatment, stoma, type of surgery, complications after surgery, and comorbidities). Age and height were centred to aid the interpretability of intercepts. The clinical factors neo-adjuvant treatment, stoma, and surgical complications were coded as not present before and at diagnosis. All fixed effects were included in the model as an interaction term with time. Only significant covariates and/or interactions were retained. Including additional interactions with time*time for the remaining covariates did not improve the model. The final model used in all analyses included the following fixed factors: time, sex, age, height, education, smoking, complications, stoma, type of surgery, comorbidities, time*time, education*time, and complications*time. The coefficient for time represents average annual linear change and the coefficient for time*time captures additional quadratic (curvilinear) change in weight in kilogrammes.

Additionally, we performed several stratified weight trajectory analyses. First, we stratified by pre-diagnosis weight change category (≥ 5% loss, stable, ≥ 5% gain) to further explore if weight gain after diagnosis differed by pre-diagnosis weight change. Second, we stratified by receipt of chemotherapy to compare weight trajectories among those treated with and without adjuvant chemotherapy. Third, as an exploratory analysis, we stratified by BMI at diagnosis to compare weight trajectories among survivors with a healthy BMI (18.5–25 kg/m^2^) and those with overweight or obesity (BMI ≥ 25 kg/m^2^). Weight trajectories were depicted based on predicted values by using the average study population, except for type of surgery in which laparoscopic surgery served as reference category. Two sensitivity analyses were performed to reduce heterogeneity between patients in the analyses stratified by chemotherapy. First by excluding patients with other adjuvant chemotherapy regimens than capecitabine combined with oxaliplatin and second by excluding patients with rectal tumours from the analyses. In the Netherlands, rectal tumours are generally not treated with adjuvant chemotherapy, which is in line with the Dutch oncological guidelines.

In all analyses, a *p* value < 0.05 was considered statistically significant. Statistical analyses were performed in SAS 9.4 (SAS Institute, Cary, NC).

## Results

Characteristics of the study population according to pre-diagnosis weight change and adjuvant chemotherapy are shown in Table 1. Participants with ≥ 5% weight gain before diagnosis were on average slightly younger, more commonly female, obese at diagnosis (BMI ≥ 30 kg/m^2^), and presenting with one or more comorbidities compared to those with either stable weight or ≥ 5% weight loss before diagnosis. Participants with ≥ 5% weight loss before diagnosis had more often a tumour located in the colon compared to those with stable weight or weight gain. Patients treated with adjuvant chemotherapy were slightly younger and had unfavourable clinical characteristics compared to patients not treated with chemotherapy; other characteristics, such as BMI, were similar between the two groups.

**Table 1:** Clinical and personal characteristics of 1152 non-metastatic colorectal cancer patients according to pre-diagnosis weight change and adjuvant chemotherapy^1^

		Weight change in the 2 years before diagnosis				Adjuvant chemotherapy		
	Overall^2^	Loss (≥ 5%)	Stable (−5% to 5%)	Gain (≥ 5%)	*p* value weight change group	No	Yes	*p* value chemo therapy
*N* (%)	1152 (100%)	279 (25%)	788 (69%)	69 (6%)		844 (75%)	282 (25%)	
Sex					< 0.001			0.21
Men	737 (64%)	179 (64%)	527 (67%)	19 (28%)		547 (65%)	171 (61%)	
Age at diagnosis (mean ± SD), years	66 ± 9	66 ± 9	66 ± 8	63 ± 11	0.029	67 ± 9	63 ± 8	< 0.001
BMI at diagnosis (mean ± SD), kg/m^2^	26.5 ± 4.0	26.0 ± 4.0	26.5 ± 3.9	29.2 ± 4.1	< 0.001	26.5 ± 3.9	26.6 ± 4.3	0.66
BMI at diagnosis, kg/m^2^					< 0.001			0.64
< 18.5	10 (1%)	3 (1%)	6 (1%)	0 (0%)		6 (1%)	4 (1%)	
18.5–25	447 (39%)	130 (47%)	301 (38%)	13 (19%)		331 (39%)	107 (38%)	
25–30	497 (43%)	110 (39%)	352 (45%)	27 (39%)		362 (43%)	122 (43%)	
30–35	161 (14%)	27 (10%)	107 (14%)	23 (33%)		120 (14%)	37 (13%)	
> 35	37 (3%)	9 (3%)	22 (3%)	6 (9%)		25 (3%)	12 (4%)	
Education level					0.061			0.37
Low	505 (44%)	133 (48%)	330 (42%)	36 (52%)		383 (45%)	115 (41%)	
Medium	277 (24%)	58 (21%)	198 (25%)	20 (29%)		195 (23%)	72 (26%)	
High	365 (32%)	87 (31%)	260 (33%)	13 (19%)		262 (31%)	95 (34%)	
Smoking at diagnosis					0.005			0.27
Yes	133 (11%)	47 (17%)	73 (9%)	11 (16%)		102 (12%)	25 (9%)	
Former	682 (59%)	166 (60%)	468 (59%)	40 (58%)		498 (59%)	168 (59%)	
Never	334 (29%)	67 (24%)	247 (32%)	18 (26%)		240 (28%)	89 (32%)	
Tumour stage					0.26			< 0.001
I	299 (26%)	60 (22%)	218 (28%)	19 (28%)		297 (35%)	–	
II	350 (30%)	96 (34%)	229 (29%)	19 (28%)		311 (37%)	27 (10%)	
III	503 (44%)	123 (44%)	341 (43%)	31 (45%)		236 (28%)	255 (90%)	
Tumour location					0.038			< 0.001
Colon	778 (68%)	206 (74%)	517 (66%)	45 (65%)		499 (59%)	259 (92%)	
Rectum	374 (32%)	73 (26%)	271 (34%)	24 (35%)		345 (41%)	23 (8%)	
Adjuvant chemotherapy					0.18			–
Yes	282 (24%)	80 (29%)	184 (23%)	16 (23%)		–	282 (100%)	
No	844 (73%)	192 (69%)	587 (74%)	52 (75%)		844 (100%)	–	
Adjuvant chemotherapy regimen					0.94			–
Capecitabine + oxaliplatin	214 (19%)	61 (22%)	140 (18%)	12 (17%)		–	214 (76%)	
Capecitabine	37 (3%)	12 (4%)	22 (3%)	2 (3%)		–	37 (13%)	
Other	7 (1%)	2 (1%)	5 (1%)	0 (0%)		–	7 (2%)	
Neo-adjuvant treatment					0.34			< 0.001
Yes	270 (23%)	57 (20%)	189 (24%)	19 (28%)		250 (30%)	15 (5%)	
No	882 (77%)	222 (79%)	599 (76%)	50 (72%)		594 (70%)	267 (95%)	
Stoma					0.061			< 0.001
Yes	340 (30%)	67 (24%)	245 (31%)	22 (32%)		309 (37%)	25 (9%)	
No	783 (68%)	207 (74%)	521 (66%)	46 (67%)		508 (60%)	255 (90%)	
Surgery					0.054			0.67
Laparoscopic	725 (63%)	162 (58%)	512 (65%)	38 (55%)		538 (64%)	177 (62%)	
Conversion	73 (6%)	18 (6%)	46 (6%)	9 (13%)		51 (6%)	21 (7%)	
Open	303 (26%)	84 (30%)	198 (25%)	19 (28%)		213 (25%)	76 (27%)	
Complications after surgery					< 0.001			< 0.001
Yes	323 (28%)	96 (35%)	195 (25%)	26 (37%)		257 (30%)	55 (20%)	
No	787 (68%)	170 (61%)	569 (72%)	40 (58%)		553 (66%)	220 (78%)	
Comorbidity					0.023			0.018
Yes	773 (67%)	182 (65%)	524 (67%)	56 (81%)		581 (69%)	173 (61%)	
No	370 (32%)	97 (35%)	256 (32%)	12 (17%)		256 (30%)	107 (38%)	

^1^Some counts do not add to totals because of missing data

^2^Includes 16 participants with missing pre-diagnosis weight and 26 with missing chemotherapy status

Compared to pre-diagnosis weight, mean weight change was −0.8 (95% CI −1.1, −0.4) kg over the 4-year period (Table 2). Over this total period, weight change was < 5% for the majority of people (66%), while 14% of all patients experienced pre-to-post diagnosis weight gain of ≥ 5% and 20% experienced weight loss of ≥ 5%. When only the 2 years post-diagnosis were taken into account, mean weight change in the 2 years after diagnosis was + 1.2 (95%CI 0.9, 1.5) kg.

**Table 2:** Two-year post-diagnosis and 4-year pre-to-post diagnosis weight changes by pre-diagnosis weight change groups and by adjuvant chemotherapy^1^

		Weight change during 2 years before diagnosis			Adjuvant chemotherapy	
	Overall	Loss (≥ 5%)	Stable (−5% to 5%)	Gain (≥ 5%)	No	Yes
*N* (%)	922 (100%)	221 (24%)	644 (70%)	57 (6%)	687 (75%)	215 (23%)
Pre-diagnosis weight change (mean (95% CI)), kg	−1.9 (−2.3, −1.6)	−8.3 (−9.1, −7,5)	−0.5 (−0.6, −0.3)	+6.2 (5.5, 6.8)	−1.9 (−2.2, −1.5)	−2.2 (−2.8, −1.5)
Pre-to-post diagnosis weight change (4 years)						
Absolute weight change (mean (95% CI)), kg	−0.8 (−1.1, −0.4)	−4.8 (−5.7, −3.9)	+0.0 (−0.3, 0.4)	+6.1 (4.5, 7.6)	−0.9 (−1.4, −0.5)	−0.1 (−0.8, 0.6)
Weight loss (%)	20	48	11	4	21	16
Weight stable (%)	66	47	76	30	65	69
Weight gain (%)	14	5	12	67	14	15
Post-diagnosis weight change (2 years)						
Absolute weight change (mean (95% CI)), kg	+1.2 (0.9, 1.5)	+3.5 (2.7, 4.3)	+0.5 (0.2, 0.8)	−0.1 (−1.7, 1.5)	+0.9 (0.5, 1.3)	+2.1 (1.5, 2.7)
Weight loss (%)	9	5	9	26	10	6
Weight stable (%)	70	54	77	55	71	67
Weight gain (%)	21	42	14	19	19	27

^1^230 participants with pre-diagnosis weight and/or 2-year post-diagnosis weight information missing omitted from table (*n* = 16 and *n* = 217, respectively)

**Fig. 1:**
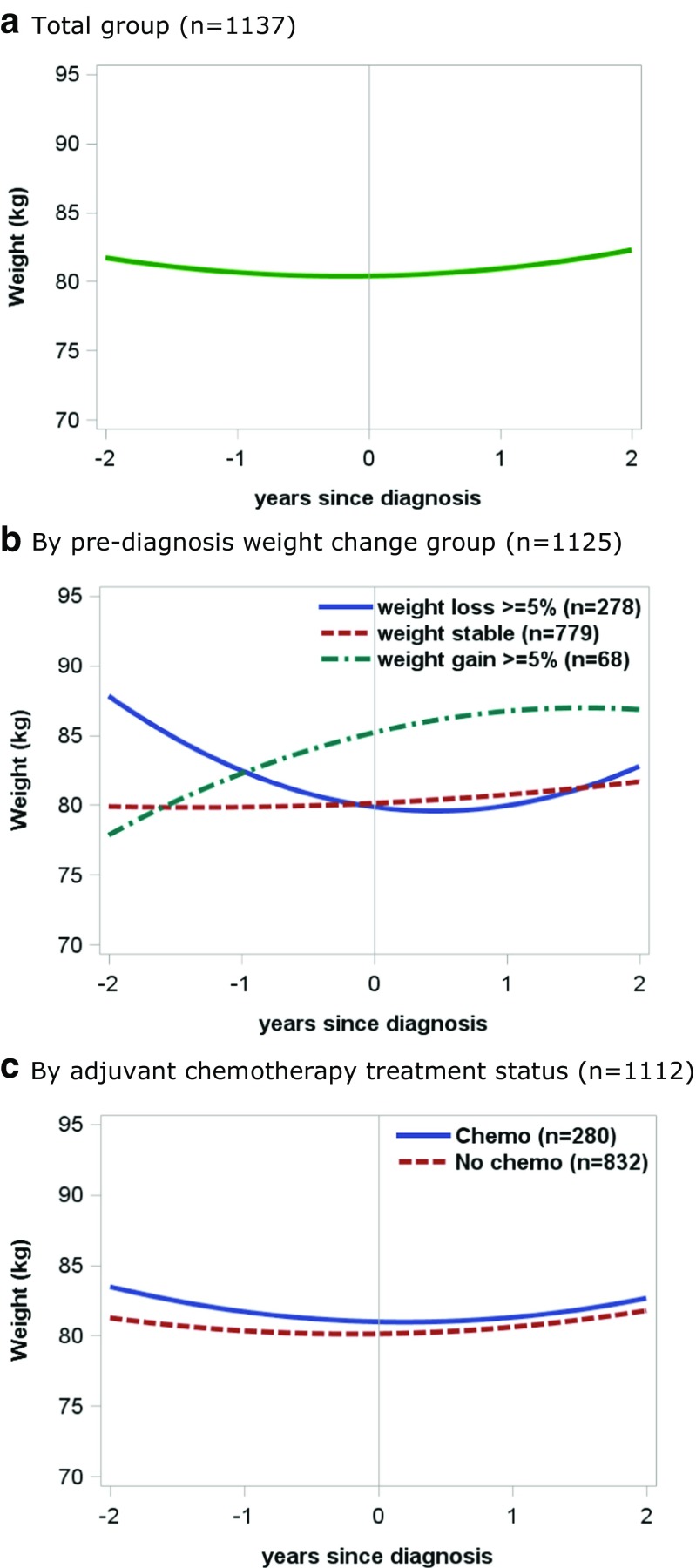
Weight trajectories from 2 years before diagnosis to 2 years after diagnosis in colorectal cancer patients (weight trajectories were based on predicted values from mixed models for a population with laparoscopic surgery and of average age, height, education, sex, smoking status, complications, stoma, type of surgery, and comorbidities). **A.** Total group (*n* = 1137) **B.** By pre-diagnosis weight change group (*n* = 1125) **C.** By adjuvant chemotherapy treatment status (*n* = 1112)

The estimated 4-year weight trajectory in the entire cohort is presented in Fig. 1A. The full model showed a clear positive quadratic relationship of weight changes in the entire cohort (*p* < 0.001), but no linear effect was present (+ 0.04 kg annual weight gain, *p* = 0.68). In other words, weight decreased before diagnosis while weight increased after diagnosis. Overall, weight 2 years after diagnosis was similar to weight 2 years before diagnosis.

To explore if post-diagnosis weight trajectories differed by pre-diagnosis weight change, we stratified the weight trajectory analyses by pre-diagnosis weight change. A mean gain in body weight after diagnosis was most prominent in the group that had lost weight before diagnosis (Fig. 1B; Table 2). In this group, 42% gained weight after diagnosis and this proportion was much larger than that seen for the pre-diagnosis weight stable and weight gain groups (14% and 19%, respectively; Table 2). In absolute numbers, post-diagnosis weight gain was on average + 3.5 (95% CI 2.7, 4.3) kg in the group that had lost weight pre-diagnosis. However, taking the 2 years before diagnosis into account, mean weight change was −4.8 (95% CI −5.7, −3.9) kg in this group. On average, clinically relevant weight change after diagnosis was absent when pre-diagnosis weight was stable or when pre-diagnosis weight gain ≥ 5% was present.

Weight trajectories were similar for those treated with and without adjuvant chemotherapy (Fig. 1C; Table 2). In both groups, overall weight 2 years after diagnosis was similar to overall weight 2 years before diagnosis. Sensitivity analyses excluding patients with other adjuvant chemotherapy regimens than capecitabine combined with oxaliplatin or excluding patients with rectal tumours did not change the results (data not shown). Weight trajectories were similar for those with a BMI of 18.5–25 kg/m^2^ and a BMI ≥ 25 kg/m^2^ at diagnosis (data not shown).

## Discussion

We examined pre-to-post diagnosis weight trajectories among patients with non-metastatic CRC. Overall, hardly any pre-to-post diagnosis weight change was observed among CRC patients, because post-diagnosis weight gain was mainly observed in patients who lost weight before diagnosis. This was observed independent of treatment with adjuvant chemotherapy.

This was the first study that examined pre-to-post diagnosis weight changes in CRC patients, therefore we can only compare our results on post-diagnosis weight changes with previous studies. All previous studies on post-diagnosis weight change in CRC patients with non-metastatic disease showed that weight gain was more common than weight loss [1–3, 10, 11, 17], which is in line with our study. We found that 21% of patients with non-metastatic CRC experienced ≥ 5% weight gain in the first 2 years after diagnosis, which is slightly lower than the 28% reported in previous studies [1, 2]. Among patients treated with adjuvant chemotherapy, 27% of patients experienced ≥ 5% weight gain in our study. Although the proportion of patients treated with chemotherapy who experienced weight gain in the current study was lower compared with other studies (36–65%) [3, 10, 11], the mean post-diagnosis weight gain of + 2.1 kg in patients treated with chemotherapy was similar to the mean weight gain of + 2.0 kg reported in a previous study based on body weights retrieved from medical records [11]. Weight gain was seen both during and after adjuvant chemotherapy [11], although in this study we were not able to make this distinction. While previous studies focussed on post-diagnosis weight changes, the current study also included usual weight pre-diagnosis into the analysis of weight changes. Our analyses revealed that post-diagnosis weight gain was most prominent in patients who lost ≥ 5% weight before diagnosis and therefore mean pre-to-post diagnosis weight gain was absent in the overall population.

The current study was the first that compared weight changes between CRC patients treated with and without adjuvant chemotherapy. By including weight data at multiple time points during the course of the disease, both before and after diagnosis, we showed that weight trajectories were similar for those treated with and without chemotherapy. In both groups, weight 2 years post-diagnosis diagnosis did on average not surpass usual pre-diagnosis weight. However, in both groups, about 15% experienced pre-to-post diagnosis weight gain of ≥ 5%. It was unexpected that weight trajectories over the course of CRC were independent of adjuvant chemotherapy treatment. Previous studies showed that post-diagnosis weight gain was more common in studies among patients treated with adjuvant therapy than in studies that included patients irrespective of adjuvant chemotherapy (36–65% versus 28%, respectively) [1–3, 10, 11]. Our results imply that weight gain is not a common side-effect of adjuvant chemotherapy in CRC patients with non-metastatic disease.

A limitation of this study is that body weight was self-reported at each time point, perhaps leading to measurement error with regard to weight change. Cross-sectional data show that self-reported weight values are typically slightly lower than directly measured values [18], although bias may differ by weight status and gender [18, 19]. However, good-to-excellent agreement was reported for self-reported and directly measured values of body weight in studies with similar demographic characteristics to this study [20, 21]. Participants are also likely to have internal consistency in their reporting, such that the degree of underreporting will be similar each time [19]. Therefore, changes in weight may be less prone to such bias than individual weight measurements. In our study, weight 2 years prior to diagnosis was recalled while post-diagnosis weights were collected prospectively, which may decrease internal consistency. However, good-to-excellent agreement was also reported for pre-diagnosis weight recalled shortly after diagnosis and directly measured values of pre-diagnosis body weight [22]. We assume that weight 2 years before CRC diagnosis reflects usual pre-diagnosis weight, since the median time from onset of symptoms (such as weight loss) until the start of treatment is usually 4 to 5 months [23, 24]. Another limitation is that we did not have information on changes in body composition. Even when pre-to-post diagnosis weight gain is not present, post-diagnosis weight gain may still lead to an increase in fat mass with a loss in muscle mass. Future research should be done to determine how post-diagnosis weight gain affects body composition.

This study has several strengths. First, the COLON study provided an opportunity to explore weight trajectories over the course of the disease in a large group of CRC patients, since we prospectively collected weight several times after diagnosis and also had pre-diagnosis weight available. We used mixed models to examine weight trajectories over 4 years. An advantage of mixed models is that participants with incomplete weight data were still included in the analyses. Second, we had detailed treatment information available so we were able to compare weight trajectories between those treated with and without adjuvant chemotherapy. Third, we were able to adjust for many covariates that could potentially affect weight change. Although other factors, such as physical activity and physical functioning, not included in the multivariate analyses could also affect weight change. However, both the adjusted weight trajectories (Fig. 1) and the crude weight changes (Table 2) showed similar results. Lastly, the study population was representative of the total population of Dutch stage I–III CRC survivors with respect to stage of disease and location of the tumour (colon or rectum), but the proportion of females and the mean age were slightly lower as compared to the total population of CRC survivors [25, 26]. Although not perfectly comparable, we believe our findings are generalizable to the total Dutch population of stage I–III CRC survivors, but they cannot be generalised to stage IV CRC survivors.

In clinical practice, not only weight loss, but also weight gain should receive attention as is stated in the Dutch Dieticians Oncology Group guidelines for bowel cancer therapy [5]. Based on our results, weight changes should be monitored over the course of the disease in all patients, taking pre-diagnosis weight change into account. A previous study suggested that pre-to-post diagnosis weight change, weight loss as well as weight gain, may be associated with a higher mortality risk among CRC patients with non-metastatic disease [1]. In contrast, post-diagnosis weight gain did not seem to be associated with mortality risk [1, 2, 10]. Our results, together with these other studies [1, 2, 10], emphasise the importance of taking pre-diagnosis weight into account when examining weight changes in CRC patients. Our study showed that 14% of all patients experienced pre-to-post diagnosis weight gain and pre-to-post diagnosis weight gain was equally prevalent among patients treated with and without adjuvant chemotherapy. Therefore, weight gain prevention should not only be targeted at patients receiving adjuvant chemotherapy, but at all CRC patients with non-metastatic disease.

In conclusion, pre-to-post diagnosis weight change was largely absent among CRC patients with non-metastatic disease, because post-diagnosis weight gain was mainly observed in patients who lost weight before diagnosis. This was observed independent of treatment with adjuvant chemotherapy. Future studies are needed to confirm our findings and to assess how weight change relates to survival and the development of co-morbidities to provide a solid basis for future recommendations directed towards managing weight during the course of CRC.
